# Novel Multimodal Management of Post-Partum Synchronous Metastatic Pulmonary EBV-Associated Lymphoepithelioma-Like Carcinoma (LELC)—A Case Report

**DOI:** 10.3390/diagnostics11112072

**Published:** 2021-11-09

**Authors:** Montserrat Pazos, Chukwuka Eze, Kathrin Kahnert, Maria Delius, Amanda Tufman, Irene Alba-Alejandre, Marcus Unterrainer, Jens Neumann, Thomas Kirchner, Farkhad Manapov

**Affiliations:** 1 Department of Radiation Oncology, University Hospital, Ludwig Maximilian University (LMU) of Munich, Marchioninistrasse 15, 81377 Munich, Germany; Chukwuka.Eze@med.uni-muenchen.de (C.E.); Farkhad.Manapov@med.uni-muenchen.de (F.M.); 2 Member of the German Center for Lung Research, Department of Medicine V, University Hospital, LMU Munich, 81377 Munich, Germany; kathrin.kahnert@med.uni-muenchen.de (K.K.); Amanda.Tufman@med.uni-muenchen.de (A.T.); 3 Department of Gynaecology and Obstetrics, University Hospital, Ludwig Maximilian University, 80337 Munich, Germany; Maria.Delius@med.uni-muenchen.de (M.D.); Irene.Alba-Alejandre@med.uni-muenchen.de (I.A.-A.); 4 Member of the German Center for Lung Research (DZL), Comprehensive Pneumology Center Munich (CPC-M), 81377 Munich, Germany; 5 Department of Radiology, University Hospital, LMU Munich, Marchioninistr. 15, 81377 Munich, Germany; Marcus.Unterrainer@med.uni-muenchen.de; 6 Department of Nuclear Medicine, University Hospital, LMU Munich, Marchioninistr. 15, 81377 Munich, Germany; 7 Department of Pathology, University Hospital, Ludwig Maximilian University, 81377 Munich, Germany; Jens.Neumann@med.uni-muenchen.de (J.N.); Thomas.Kirchner@med.uni-muenchen.de (T.K.); 8 German Cancer Consortium (DKTK), Partner Site Munich, 80336 Munich, Germany

**Keywords:** EBV-associated lymphoepithelioma-like lung carcinoma, LELC, multi-site radiation therapy, chemoimmunotherapy, lung cancer in pregnancy

## Abstract

Primary Epstein-Barr-Virus (EBV)-associated pulmonary lymphoepithelioma-like carcinoma (LELC) is an aggressive rare cancer. Higher incidences have been observed in Asian sub-populations. Multimodal treatment paradigms have emerged as promising novel strategies in the management of advanced NSCLC. In this report, we describe the case of a 34-year-old female patient of Asian origin with a post-partum initial diagnosis of pulmonary LELC. Multimodal treatment with chemoimmunotherapy and hypofractionated irradiation to the primary tumour and main metastatic sites led to a favourable response demonstrating that radiotherapy may potentially augment anti-tumour immunity. To the best of our knowledge, this is the first case report on this novel therapy strategy of multi-site hypofractionated radiotherapy and chemoimmunotherapy for metastatic pulmonary EBV-associated LELC.

## 1. Introduction

Epstein-Barr-Virus(EBV)-associated lymphoepithelioma-like carcinoma (LELC) of the lung is an extremely rare, non-small cell lung cancer subtype accounting for approximately 0.7% of cases [1], predominantly seen in female Asian never-smokers. As of yet, there is a paucity of data on its optimal management. Contemporary reports describing treatment with immune checkpoint inhibitors (ICIs) have been published [1,2,3,4]. Promising results in the management of advanced NSCLC with the combination of chemoradioimmunotherapy portend the successful application of a multimodal approach in this setting [5].

## 2. Case Presentation

**Figure 1:**
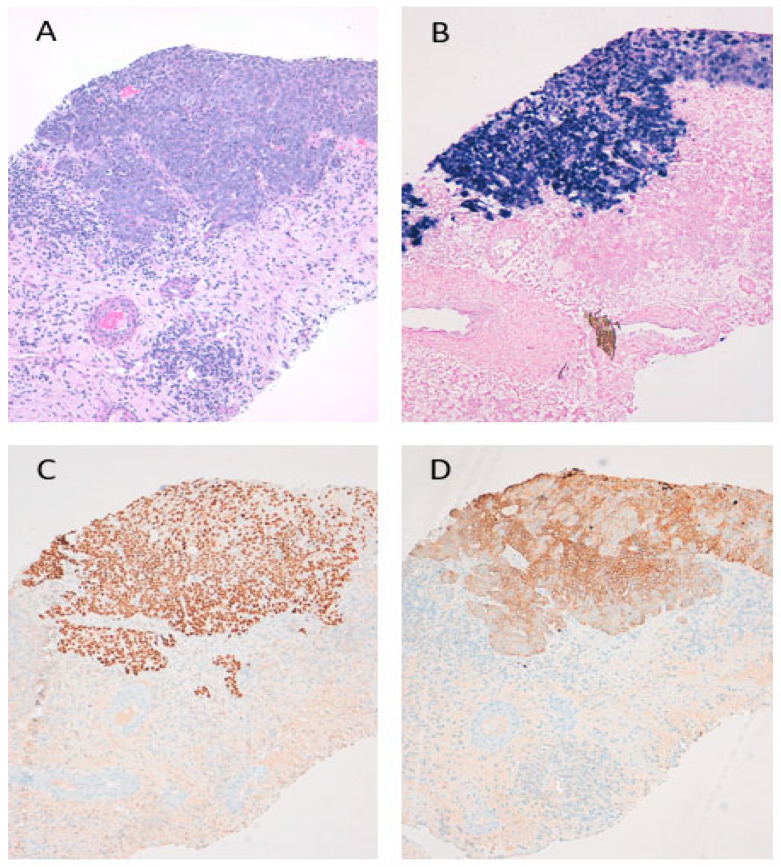
Histology reveals a poorly differentiated carcinoma with syncytial growth pattern and dense lymphocytic infiltrate (**A**). In situ hybridisation for EBV-encoded small RNA (EBER) selectively labels the carcinoma cells, while the lymphoid cells are negative (**B**), proving infection with Epstein-Barr-Virus (EBV). The tumour cells show diffuse staining for p40 (**C**) and cytokeratin 5/6 (**D**), both indicating squamous differentiation.

A 34-year-old never-smoker Asian woman was referred to our department in October 2020 after a post-partum diagnosis of metastatic pulmonary LELC. Prior to diagnosis, the patient (63 kg, 172 cm) had complained of lower back/hip pain radiating down the right leg during pregnancy. In week 35, the patient went into spontaneous labour and underwent a cesarean section. Due to pain aggravation post-partum, an MRI scan was performed, revealing multiple bone metastases with the main sites in the upper third of the right femur, sacral bone and Th8–9. A CT-guided biopsy of the bone lesion in the right femur was suggestive of secondary metastasis of poorly differentiated SCC with lymphoplasmacytic infiltrates (p40, p63, CK5/6 and EBV positive; ER/PR 30%/10%; p16, TTF-1, CK20 & GATA 3 negative). A further histopathologic evaluation confirmed LELC (Figure 1).

**Figure 2:**
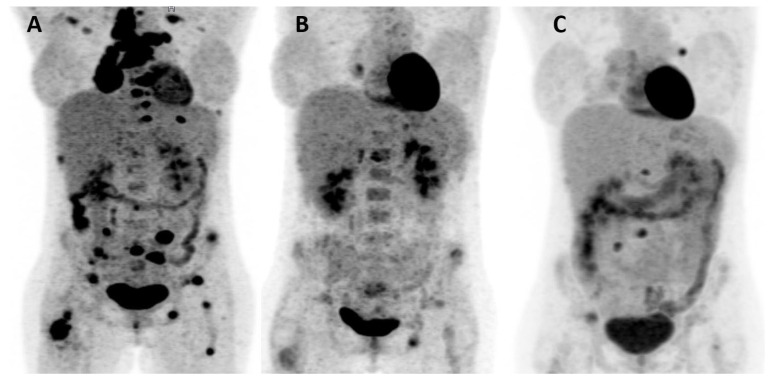
PET-CT prior to treatment (**A**), showing remarkable response after 2 cycles of chemoimmunotherapy and multi-site radiotherapy (**B**) and slight progression with a new PET-avid left hilar lymph node and a bone metastasis in L3 (**C**).

**Figure 3:**
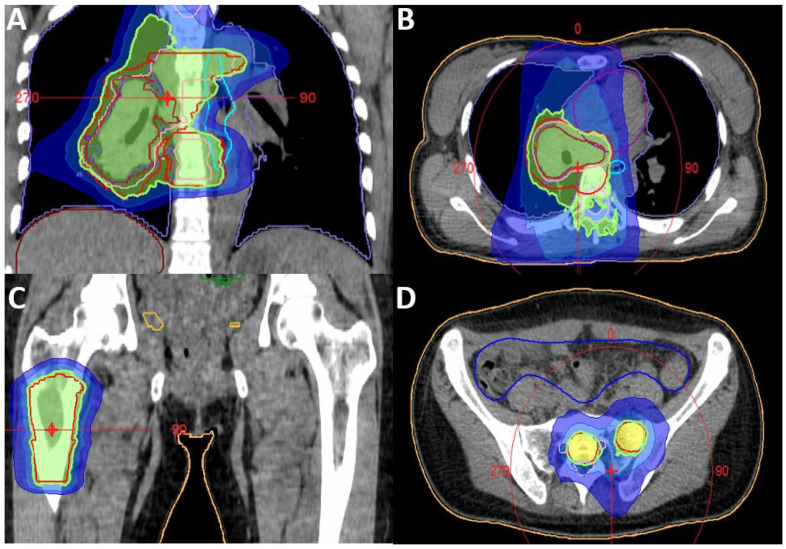
Radiotherapy volumes of “multi-site” radiotherapy of a 34-year-old woman with stage IV NSCLC: primary tumour & Th8-9 (**A**/**B**), right Femur (**C**) and sacral lesions (**D**). Dose concept for all locations: 5 × 4.0 Gy.

An ^18^F-FDG PET/CT revealed a large stenotic tumour in the right lower lobe with multiple PET-positive mediastinal lymph nodes, a satellite lesion and multiple bone metastases (Figure 2). There was no evidence of brain metastasis after performing a cranial MRI, hence cT3 cN3 cM1c (TNM 8th edition). Next-generation sequencing (OncomineTM Focus Assay, Thermo Fisher Scientific, MA, USA) found no actionable driver mutations but did find an inactivating E886X mutation in the *ATRX-gene*; the PD-L1 TPS was 10%. Following a discussion at the multidisciplinary tumour board (MTB), systemic treatment with platinum-based chemoimmunotherapy (cisplatin 75 mg/m^2^, first cycle)/carboplatin AUC5; paclitaxel 200 mg/m^2^; pembrolizumab 200 mg every three weeks (q3W) and bisphosphonates every 4 weeks (q4W) were initiated in 11/2020. Between the first two cycles, moderate hypofractionated radiotherapy was delivered to the metastases in the right femur and the primary intrathoracic tumour with the inclusion of Th8-9 in the same target volume with 5 daily fractions of 4.0 Gy to a total dose of 20.0 Gy (Figure 3). Radiotherapy was well-tolerated and alleviated the back pain, therefore, seamless radiotherapy of the PET positive metastases in the sacral bone followed (Figure 3).

A follow-up PET/CT 12/2020 after 2 cycles revealed a favourable response with a partial radiological (RECIST 1.1) and metabolic response (PMR). PET/CT after 4 cycles 01/2021 showed slight progression (PD). The patient received two further cycles and continued with pembrolizumab monotherapy from 03/2021 onwards. Another PET/CT, after 5x pembrolizumab confirmed disease progression (nodal, bone). The case was again discussed 06/2021 at MTB and, after a renewed biopsy of a new lesion of the L5 confirmed LELC, a second-line systemic therapy analogue CheckMate 9LA was recommended [6]. The first cycle was recently administered, and renewed analgetic moderately hypofractionated radiotherapy of the bone metastases in L4/5 with 20.0/4.0 Gy has now been planned.

## 3. Discussion

LELC is a rare subtype of NSCLC. Recently, Yeh at al. identified a group of EBV-associated carcinomas with a less significant lymphocytic infiltration than classic LELC and similar molecular characteristics, proposing the grouping of both entities under the term “EBV-associated pulmonary carcinoma” [7]. In the present case report, we describe the medical history of a young Asian patient with a post-partum (gravida II) and newly diagnosed EBV-associated metastatic pulmonary carcinoma. This case was distinct due to a suspected gynaecologic malignancy, for which a hormone receptor immunohistochemistry was performed revealing an ER/PR status of 30%/10%, which potentially promoted tumour growth during pregnancy, a process that has previously been described in literature [8,9,10]. To the best of our knowledge, this is the first report on first-line multimodal chemoradioimmunotherapy in LELC. Our patient displayed a rapid response to the initial treatment, demonstrating favourable response at all sites. However, the oligoprogression of three of the bone metastases was confirmed approximately six months after the start of pembrolizumab maintenance. In addition, the prognostic value of circulating EBV-DNA in pulmonary LELC has been described in [11]. In the current case, the circulating EBV-DNA was not measured. However, during the course of treatment, a bone marrow aspirate and multiple oropharyngeal washes tested negative for EBV-DNA.

Previously, a study in China was published, regarding a small retrospective analysis of metastatic/recurrent LELC treated with ICIs or chemotherapy, which demonstrated superior progression-free survival in the immunotherapy arm (15 vs. 7.9 months; *p* < 0.05) [4]. In another large LELC series, including 5/128 LELC patients who received ICIs as salvage therapy, the best outcome was a stabilization of the disease in all patients [3]. The combination of chemoimmunotherapy with irradiation to sites allows for the achievement of an accelerated objective response with rapid tumour shrinkage which may correlate with signaling pathways (DAMPs, tumour antigens and neoantigens) and a stimulation of the adaptive immune system to promote an enhanced anti-tumour immune response. The rationale for using multiple/all site irradiation in order to enhance anti-tumour responses has been documented by Brooks and Chang [12]. Furthermore, the most appropriate metastatic site for irradiation, as the one that is most likely to induce immunogenic cell death, remains unclear [13]. In the current case, irradiation to the main metastatic locations was performed, leading to a partial radiological and metabolic response. The metabolic response was recently found to be a survival prognosticator in advanced NSCLC [14,15].

## 4. Conclusions

To the best of our knowledge, this is the first report on multimodal therapy for metastatic pulmonary EBV-associated LELC for moderately hypofractionated radiotherapy to the primary tumour and main metastatic sites. This treatment combination is promising and could be a better avenue than chemo-immunotherapy alone for highly selected patients (young patients, favourable PS).

## Data Availability

Not applicable.
